# Diffusion-mediated nuclear spin phase decoherence in cylindrically porous materials

**DOI:** 10.1016/j.jmr.2016.05.007

**Published:** 2016-08

**Authors:** Michael J. Knight, Risto A. Kauppinen

**Affiliations:** a School of Experimental Psychology, University of Bristol, 12A Priory Road, Bristol BS8 1TU, United Kingdom; b Clinical Research and Imaging Centre, University of Bristol, 60 St Michael’s Hill, Bristol BS2 8DX, United Kingdom

**Keywords:** Coherence lifetime, Anisotropy, Relaxation, Porous media

## Abstract

In NMR or MRI of complex materials, including biological tissues and porous materials, magnetic susceptibility differences within the material result in local magnetic field inhomogeneities, even if the applied magnetic field is homogeneous. Mobile nuclear spins move though the inhomogeneous field, by translational diffusion and other mechanisms, resulting in decoherence of nuclear spin phase more rapidly than transverse relaxation alone. The objective of this paper is to simulate this diffusion-mediated decoherence and demonstrate that it may substantially reduce coherence lifetimes of nuclear spin phase, in an anisotropic fashion. We do so using a model of cylindrical pores within an otherwise homogeneous material, and calculate the resulting magnetic field inhomogeneities. Our simulations show that diffusion-mediated decoherence in a system of parallel cylindrical pores is anisotropic, with coherence lifetime minimised when the array of cylindrical pores is perpendicular to *B*_0_. We also show that this anisotropy of coherence lifetime is reduced if the orientations of cylindrical pores are disordered within the system. In addition we characterise the dependence on *B*_0_, the magnetic susceptibility of the cylindrical pores relative to the surroundings, the diffusion coefficient and cylinder wall thickness. Our findings may aid in the interpretation of NMR and MRI relaxation data.

## Highlights

• Porous systems create small inhomogeneous magnetic fields within an applied field.
• Diffusion through this field reduces coherence lifetimes in NMR and MRI.
• Changing the orientation of the system changes the inhomogeneous field.
• This coherence lifetime anisotropy may help study porous systems.
• This includes *in vivo* systems.

## Graphical abstract

**Figure:**
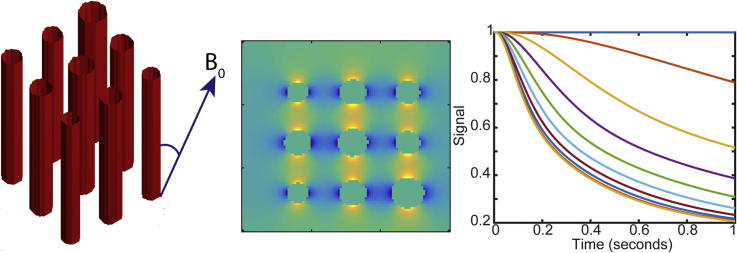

## 1 Introduction

In both a nuclear magnetic resonance (NMR) and magnetic resonance imaging (MRI) experiment, a signal is created by the generation of coherence of nuclear spin phase by radiofrequency (RF) fields. This coherence is subsequently lost and spin phases once more randomised, the system thereby returning to a state in which its entropy is maximised. The loss of spin phase coherence may occur by several means. The stochastic modulation of the nuclear spin Hamiltonian, due to Brownian motion (mainly rotational diffusion) as well as internal bond vector motions, results in stochastically fluctuating magnetic fields [1]. Such randomly changing fields are due to spin interactions, such as the chemical shift anisotropy (CSA), nuclear spin dipolar couplings, and quadrupolar couplings (for *I* > 1/2). Stochastically varying fields resulting from stochastic modulation of the nuclear spin Hamiltonian are the cause of nuclear spin *relaxation*, with the transverse relaxation time constant having the conventional abbreviation *T*_2_. As such we consider a relaxation process to be one involving stochastic modulation of the nuclear spin Hamiltonian, concomitantly returning a spin system to the equilibrium state. Relaxation is normally described using Redfield theory [2]. However, relaxation is not the only means by which spin phase coherence is lost. If nuclear spins experience a distribution of magnetic fields, either statically or dynamically across a sample, for reasons other than a stochastically varying Hamiltonian, spin phase coherence will be lost, yet the process may not properly be labelled as relaxation. A system may also therefore be characterised by a *coherence lifetime* shorter than its *T*_2_. Causes for the decoherence which are not relaxation include chemical exchange, static dephasing due to magnetic field inhomogeneities, and translational diffusion through magnetic field inhomogeneities. It is the latter phenomenon that is the subject of the current paper.

In an inhomogeneous material containing internal compartments with different magnetic susceptibilities, the compartments, which we label as “magnetic field perturbers”, result in small magnetic fields in response to the material being exposed to a strong applied magnetic field. This situation is the case in both NMR and MRI experiments of porous materials permeated by some other material, or of biological tissues. A mobile nuclear spin moving through the material (by translational diffusion or other mechanisms) experiences a distribution of magnetic fields even if the applied field is homogeneous. The ensemble of nuclear spins therefore loses coherence of phase more rapidly than due to relaxation processes alone.

A similar effect is exploited in both NMR and MRI diffusion measurements by the application of a strong pulsed linear field gradient [3]. Any coherence lost, and which cannot be refocussed by a 180° radiofrequency (RF) pulse or recalled by a pulsed field gradient causing a counter-translation through *k*-space may be attributed to translational diffusion. Classical formalisms of the effects of translational diffusion in the presence of linear field gradients on spin dynamics are well known, both for isotropic and anisotropic diffusion [3], [4], [5]. These form the basis of the theory section to come.

Our objective in the current work was to simulate the effects of cylindrical magnetic field perturbers as causes of diffusion-mediated decoherence. We will review the relevant theory, developing general expressions, and present simulations of nuclear spin dephasing due to translational diffusion through magnetic field inhomogeneities arising due to cylindrical magnetic field perturbers with different magnetic susceptibility from their surroundings.

### 1.1 Relation to similar work

The effects of spherical magnetic field perturbers on diffusion-mediated decoherence have been considered before [6]. However, no consideration in that work was given to the anisotropy of coherence lifetime, nor could the state of order of the system be considered as the field perturbers were limited to a spherical model. There has also been work to characterise the decoherence of nuclear spin phase in the static dephasing regime [7], [8], which has related decoherence to susceptibility differences, but diffusion-mediated decoherence was not incorporated into the latter formalisms.

Parallel and individual cylindrical models have previously been used to model gradient-echo phase contrast in the white matter of the brain [9], [10]. The objective in that work, however, was to explain phase contrast in MRI, and no consideration was given to diffusion-mediated decoherence, despite some overlap in conceptual model design. Similar concepts have recently been used to explain anisotropic gradient-echo $T_{2}^{*}$ (which does not use refocussing pulses and therefore contains static-dephasing susceptibility-dominated contributions) in the brain [11].

However, there has been little interest at quantifying the contributions to coherence lifetime due to diffusion-mediated decoherence. Nonetheless, the dependence of diffusion-mediated decoherence on the microscopic arrangement of structures within the system may make it a useful phenomenon. There are a variety of systems in which coherence lifetime and/or relaxation anisotropy is known to exist (though not necessarily separable with ease). *In vivo*, coherence lifetime is known to be anisotropic in cartilage [12], [13], [14] and has more recently been observed to be anisotropic in the brain [15] and peripheral nervous system [16]. To what extent the mechanisms modelled here may have a role is not yet clear.

## 2 Theory

### 2.1 Diffusion in the presence of an arbitrary magnetic field inhomogeneity

We present a classical description, therefore limited to uncoupled spin-1/2 nuclei, based on the work of Torrey [4] and later work by Stejskal and Tanner [5]. The Bloch–Torrey equation for transverse magnetisation including anisotropic translational diffusion, in the laboratory frame of reference, is:

(1) $$\frac{\partial }{\partial t}M^{+}(t\text{,}x)=(-i\omega_{0}-i\omega_{\mathrm{cs}}-i\Delta \omega (x)-R_{2}+\nabla \cdot D\nabla )M^{+}(t\text{,}x)$$

Here, $M^{+}(t\text{,}x)$ is the complex-valued transverse magnetisation as a function of time *t* and spatial coordinate **x**, $\omega_{0}$ is the Larmor frequency, $\omega_{\mathrm{cs}}$ is the isotropic part of the chemical shift anisotropy tensor, Δω(x) is a frequency difference function describing the magnetic field inhomogeneity, $R_{2}$ is the transverse relaxation rate constant, ∇ is the gradient operator, and D the translational diffusion tensor. This is immediately simplified by working in a frame rotating at the chemically shifted Larmor frequency:

(2) $$\frac{\partial }{\partial t}M_{R}^{+}(t\text{,}x)=(-i\Delta \omega (x)-R_{2}+\nabla \cdot D\nabla )M^{+}(t\text{,}x)$$

where $M_{R}^{+}(t\text{,}x)$ is the transverse magnetisation in a frame of reference rotating at the Larmor frequency, and in the same sense as Larmor precession. Following a similar approach to Torrey and others, if we assume relaxation to be isotropic and independent of space, we can factor its effects out as an exponential dampening:

(3) $$\frac{\partial }{\partial t}\psi (t\text{,}x)=(-i\Delta \omega (x)+\nabla \cdot D\nabla )\psi (t\text{,}x)$$

where

(4) $$M_{R}^{+}(t\text{,}x)=\psi (t\text{,}x)\exp(-R_{2}t)$$

In other words, transverse magnetisation may be represented by a product of a purely time-dependent function $\exp(-R_{2}t)$ and a function of time and space ψ(t,x). Now we need only solve for ψ(t,x). This may be factorised into a product of two terms:

(5) ψ(t,x)=A(t,x)exp(iρΔω(x)t)

Note that we have introduced the coherence order ρ, similarly to recent modern treatments of the effects of diffusion on the NMR signal [17]. This allows us to account for refocussing pulses. This factorisation treats ψ(t,x) as being a product of a periodic term exp(iρΔω(x)t) and some as-yet-unknown term A(t,x) which includes damping due to diffusion. However, if the frequency difference function Δω(x) (and therefore magnetic field inhomogeneity) is non-linear in space, A(t,x) is also a function of space – a point we shall return to shortly.

Differentiating ψ(t,x) with respect to time:

(6) $$\frac{\partial }{\partial t}\psi (t\text{,}x)=\frac{\partial }{\partial t}[A(t\text{,}x)\exp(i\rho \Delta \omega (x)t)]=\frac{\partial A(t\text{,}x)}{\partial t}\exp(i\rho \Delta \omega (x)t)+A(t\text{,}x)i\rho \Delta \omega (x)\exp(i\rho \Delta \omega (x)t)$$

We also have, by substituting our assumed solution (5) into (3):

(7) $$\frac{\partial }{\partial t}\psi (t\text{,}x)=(i\rho \Delta \omega (x)+\nabla \cdot D\nabla )\psi (t\text{,}x)=i\rho \Delta \omega A(t\text{,}x)\exp(i\rho \Delta \omega (x)t)+\nabla \cdot D\nabla [A(t\text{,}x)\exp(i\rho \Delta \omega (x)t)]$$

Evaluating the derivatives (see Appendix A for more details) and dropping the variables upon which terms depends gives:

(8) $$\nabla \cdot D\nabla [A\exp(i\rho \Delta \omega t)]=[\nabla \cdot D\nabla A+i\rho t[\nabla \Delta \omega \cdot D\nabla A+\nabla A\cdot D\nabla \Delta \omega ]+A(i\rho t\nabla \cdot D\nabla -\rho^{2}t^{2}\nabla \Delta \omega \cdot D\nabla )]\exp(i\rho \Delta \omega t)$$

Substituting into (7)

(9) $$\frac{\partial }{\partial t}\psi (t\text{,}x)=i\rho \Delta \omega A(t\text{,}x)\exp(i\rho \Delta \omega (x)t)+[\nabla \cdot D\nabla A+i\rho t[\nabla \Delta \omega \cdot D\nabla A+\nabla A\cdot D\nabla \Delta \omega ]+A(i\rho t\nabla \cdot D\nabla -\rho^{2}t^{2}\nabla \Delta \omega \cdot D\nabla )]\exp(i\rho \Delta \omega t)$$

By Eq. (9), (6), after some simple manipulation we obtain:

(10) $$\frac{\partial A}{\partial t}=\nabla \cdot D\nabla A+i\rho t[\nabla \Delta \omega \cdot D\nabla A+\nabla A\cdot D\nabla \Delta \omega ]+A(i\rho t\nabla \cdot D\nabla -\rho^{2}t^{2}\nabla \Delta \omega \cdot D\nabla )\Delta \omega$$

As shown in Appendix A, this can also be expressed concisely as:

(11) $$\frac{\partial A}{\partial t}=\exp(-i\rho \Delta \omega t)\nabla \cdot D\nabla [A\exp(i\rho \Delta \omega t)]$$

Recalling that *A* is essentially the diffusion damping term, the problem is solved once Eqs. (10), (11) has been solved for *A*. Eq. (10), and its concise form (11) therefore provide general expressions for classical descriptions of transverse magnetisation inclusive of anisotropic or isotropic translational diffusion in the presence of an arbitrary magnetic inhomogeneity expressed by a frequency difference function Δω(x). It is valid so long as one is not considering spin interactions or exchange. It is not, however, particularly practical, since its complexity makes general solutions challenging. If instead we introduce the simplifying assumption that the spatial dependence of *A* is locally linear on the length scale of diffusion, or at least much shallower than that of Δω, then *A* may be considered a function of time only. Then we have:

(12) $$\frac{\mathrm{dA}}{\mathrm{dt}}=A(i\rho t\nabla \cdot D\nabla -\rho^{2}t^{2}\nabla \Delta \omega \cdot D\nabla )\Delta \omega$$

The solution is straightforward:

(13) $$A(t)=A_{0}\exp\left\{\frac{i\rho t^{2}}{2}[\nabla \cdot D\nabla ]\Delta \omega (x)\right\}\exp\left\{-\frac{\rho^{2}t^{3}}{3}[\nabla \Delta \omega (x)\cdot D\nabla ]\Delta \omega (x)\right\}$$

where *A*_0_ is the initial signal intensity. The total signal amplitude, including *R*_2_ (transverse) relaxation, is obtained by integrating this function over space:

(14) $$S(t)=\left|\int A_{0}\exp\left\{\frac{i\rho t^{2}}{2}[\nabla \cdot D\nabla ]\Delta \omega (x)\right\}\exp\left\{-\frac{\rho^{2}t^{3}}{3}[\nabla \Delta \omega (x)\cdot D\nabla ]\Delta \omega (x)\right\}dx\right|\exp(-R_{2}t)$$

In the damping term, note that the coherence order is always positive (appears in the second power) and that the signal is damped with the cube of time and linearly with components of the diffusion tensor. In the oscillating (phase) term, the coherence order may be variable (but is required to be piecewise constant with time), and the signal evolves with the square of time and linearly with components of the diffusion tensor. Since the coherence order changes between −1 and +1 between refocussing pulses, we can expect a spin-echo sequence to refocus this term, but to have additional dephasing in gradient echo sequences. The formula above evaluates to the well-known results of Stejskal and Tanner, if we let

(15) Δω(x)=γG·x

With **G** a magnetic field gradient vector and γ the gyromagnetic ratio for the nucleus under observation.

### 2.2 Field inhomogeneity function in cylindrically porous materials

Now, we require a form of the frequency difference function (proportional to magnetic field inhomogeneity), arising due to interfaces between materials of different magnetic susceptibility. We are interested in the case of cylindrical structures, which arise in various materials including biological tissues. For example, capillaries and veins (containing paramagnetic deoxyhaemoglobin) and the walls of myelinated axons (which are diamagnetic) may be modelled as cylinders with different magnetic susceptibility from their surroundings. For a single hollow cylinder with an infinitely thin wall, where the material inside the cylinder has a different susceptibility from the material outside, we have [9]:

(16) $$\Delta \omega (x)=\left\{\begin{array}{cc} \frac{\omega_{0}\chi \sin^{2}\theta \cos2\phi }{2}\left(\frac{r_{c}^{2}}{r^{2}}\right)\text{,} & r⩾r_{c}\\ 0\text{,} & r<r_{c} \end{array}\right)$$

where *ω*_0_ is the Larmor frequency, θ is the polar angle between the long axis of the cylinder and *B*_0_, and the coordinates ϕ, *r* represent position in a cylindrical system with the *z*-axis parallel to the cylinder long axis and *B*_0_ defined in the *xz* plane. χ is the susceptibility difference (with the susceptibility tensor assumed isotropic) between the outside and inside of the cylinder, and *r*_c_ is the cylinder radius. In the case of an array of hollow cylinders, this may be summed:

(17) $$\Delta \omega (x)=\underset{j}{\sum }\left\{\begin{array}{cc} \frac{\omega_{0}\chi_{j}\sin^{2}\theta_{j}\cos2\phi_{j}}{2}\left(\frac{r_{\mathrm{cj}}^{2}}{r^{2}}\right)\text{,} & r⩾r_{\mathrm{cj}}\\ 0\text{,} & r<r_{\mathrm{cj}} \end{array}\right)$$

Now, cylinders are enumerated by *j*.

We can also take account of finite wall thickness, in which case the above equation becomes:

(18) $$\Delta \omega (x)=\underset{j}{\sum }\left\{\begin{array}{cc} \frac{\omega_{0}\chi_{j}}{2}\sin^{2}\theta_{j}\cos2\phi_{j}\left(\frac{r_{\mathrm{cj}}^{2}}{r^{2}}\right)\text{,} & r⩾r_{\mathrm{cj}}\\ \frac{\omega_{0}\chi_{j}}{2}\left(\cos^{2}\theta_{j}-\frac{1}{3}-\sin^{2}\theta_{j}\cos2\phi_{j}\left(\frac{r_{\mathrm{cj}}^{2}-r_{\mathrm{Lj}}^{2}}{r^{2}}\right)\right)\text{,} & r_{\mathrm{Lj}}⩽r<r_{\mathrm{cj}}\\ 0\text{,} & r<r_{\mathrm{Lj}} \end{array}\right)$$

Once we have chosen geometry for a set of cylinders, Eqs. (16), (17) may be used to calculate a frequency difference function, and Eq. (13) applied to calculate the resulting decoherence of nuclear spin phase. We created a Matlab class capable of calculating the frequency difference function for any arbitrary geometry of any number of cylindrical field perturbers, based of Eqs. (17), (18), and which then determined the complex-valued evolution of the magnetic resonance signal due to diffusion only according to Eq. (14). We used Matlab version 2015b for all our simulations.

### 2.3 Correlation functions for relaxation due to translational diffusion

A correlation function for some arbitrary spin interaction may be expressed as

(19) $$C_{\mathrm{qq}}(t)=\int F^{\left(q\right)}(x_{0})F^{\left(-q\right)}(x)P(x_{0})P(x\left|x_{0}\right))dxdx_{0}$$

Here, the functions $F^{\left(\pm q\right)}$ are the spatial parts of the Hamiltonian for the spin interaction of interest (e.g. CSA, dipolar couplings, etc.), *q* indexes the basis functions in the expansion of the Hamiltonian, $P(x_{0})$ is the probability that a spin will have coordinate **x**_0_ at t = 0 and $P(x\left|x_{0}\right))$ is the conditional probability that a spin will make a jump to coordinate **x** at time t given coordinate **x**_0_ at t = 0.

## 3 Results

### 3.1 Effects of a single cylinder

**Fig. 1:**
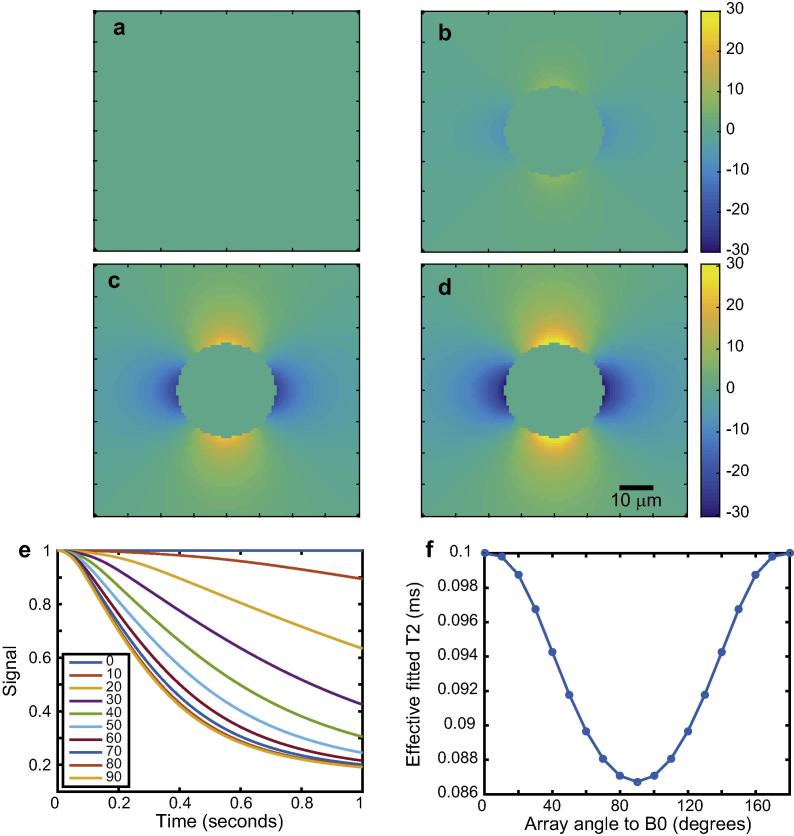
Effects of translational diffusion through magnetic field inhomogeneities caused by a single cylindrical pore of 30 μm diameter. Both the cylinder and surroundings contain solvent with ^1^H nuclei which are observed. Panels a–d show the frequency difference function when the cylinder is inclined at angles of 0°, 30°, 60° and 90° relative to *B*_0_. All panels a–d share the scale bars of b and d, showing frequencies in Hz. Panel e shows the loss of nuclear spin phase due to diffusion through the magnetic field inhomogeneities at a variety of cylinder angles relative to *B*_0_. Panel f shows “effective *T*_2_” values obtained by damping the curves in panel e with a *T*_2_ of 0.1 s, then fitting a mono-exponential function to the total resulting decoherence function.

As a first demonstration of the effects of cylindrical field perturbers whose magnetic susceptibility is different from their surroundings, we consider a single cylinder with an impermeable and infinitely thin wall. This may model a cylindrical pore in a material. As in all our simulations, for the sake of simplicity we assume translational diffusion to be isotropic, and likewise the magnetic susceptibility to be isotropic. We also assume that the pores, their walls (in later simulations) and the surroundings contain ^1^H-containing solvent which is observed, such that the Larmor frequency appearing in Eqs. (16), (17), (18), (19) is that of ^1^H. Fig. 1 shows simulations of what we can expect for a single cylinder. Simulations were performed assuming a *B*_0_ field of 3 T (a common field strength for modern clinical MRI scanners), an isotropic susceptibility difference of 0.5 ppm, an isotropic diffusion coefficient of 1 μm^2^/s and using a single cylinder of radius 30 μm in a cube of 80 × 80 × 80 μm^3^. From panels 1a–d, we see that as the cylinder is inclined relative to the applied field, it creates an inhomogeneous magnetic field in response, plotted as frequency difference maps at a representative slice (this system has cylindrical symmetry). Clearly, nuclear spins will diffuse through a magnetic field changing more rapidly in space as the angle between the longitudinal axis of the cylinder and the applied field approaches 90°. As shown in Fig.1e, dephasing is greatest at 90°. This is *coherence lifetime anisotropy*. We have also calculated coherence lifetime or “effective *T*_2_” values. Although the diffusion-mediated decoherence is plainly non-exponential, it remains common practice in NMR and MRI to consider overall decoherence to be exponential, so we have damped the functions in Fig.1e with a relaxation time *T*_2_ of 0.1 s, then fitted a mono-exponential function to the total decay functions to yield effective coherence lifetimes, plotted in Fig.1f. We can then more clearly see the anisotropy of coherence lifetime; as the system is physically rotated with respect to B_0_, the coherence lifetime, defined as the effective time constant with which nuclear spin phase coherence is irretrievably lost (and cannot be refocussed), is also changed. We also attempted to extract model-free coherence lifetimes by calculating the second central moment of the relaxation-damped simulations, but found the numerical stability of the (model-dependent) exponential method to be superior. This is explained in detail with examples in the Supplementary Material.

### 3.2 Effects of *B*_0_

**Fig. 2:**
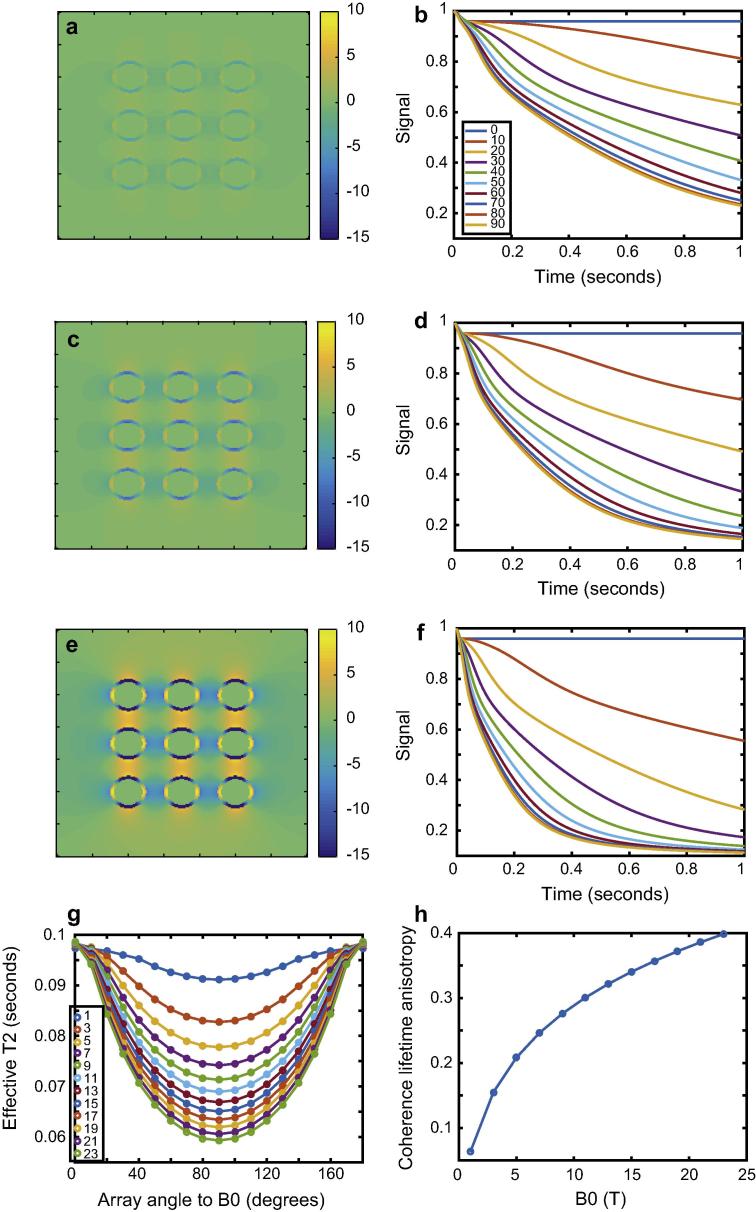
The effects of *B*_0_ on nuclear spin phase decoherence in cylindrically porous media. Panels a, c, and e show frequency difference maps calculated for an array for cylindrical field pertubers inclined at 90° relative to *B*_0_ with *B*_0_ = 3, 7 and 19 T respectively, Panels b, d, and f show the corresponding diffusion-mediated decoherence of the signal amplitude. Panel e shows the effective coherence lifetime as a function of orientation under the restrictive assumption that there is an inherent *T*_2_ of 0.1 s at all fields. Panel h shows the coherence lifetime anisotropy as a function of *B*_0_.

We next performed a simulation using a more complex model of multiple cylindrical pores with walls of finite thickness, in which the walls were composed of a material with different magnetic susceptibility from the surroundings and lumen (the latter two regions having the same magnetic susceptibility). As before, the simulations treated all regions as containing solvent with ^1^H nuclei in equal concentration in all regions to be observed. Such a system may be used to model myelinated axons of white matter fibres in the brain. We created an array of 9 cylinders, each of outer radius 0.5 μm and inner radius 0.1 μm. The susceptibility difference between the wall and surroundings/lumen was 0.05 ppm, and the diffusion coefficient 0.7 μm^2^/s, similar to human brain parenchyma. We varied the *B*_0_ field from 1 T to 23 T, covering the typical range of field strengths available from commercial MRI and NMR systems. The results are shown in Fig. 2. The coherence lifetime, as before, was obtained heuristically by damping the diffusion-attenuated signal by an exponential function assuming a *T*_2_ of 0.1 s regardless of field strength and orientation. We then defined the coherence lifetime anisotropy parameter as:

(20) $$C=\frac{T_{2}^{0}-T_{2}^{90}}{T_{2}^{0}}$$

where $T_{2}^{0}$ and $T_{2}^{90}$ denote the coherence lifetime with the system oriented at 0° and 90° relative to *B*_0_ respectively. It is clear that diffusion-mediated decoherence becomes more severe as *B*_0_ increases. Although the same pattern of coherence lifetime anisotropy is seen regardless of field strength, the *C* parameter increases with increasing field. This is a reasonable result given that the Larmor frequency appears in Eqs. (16), (17), (18). We can expect that magnetic field gradients are steeper at higher fields, such that a greater dispersion in phase is imposed across the same region of space by a higher *B*_0_.

### 3.3 Effects of diffusion coefficient

**Fig. 3:**
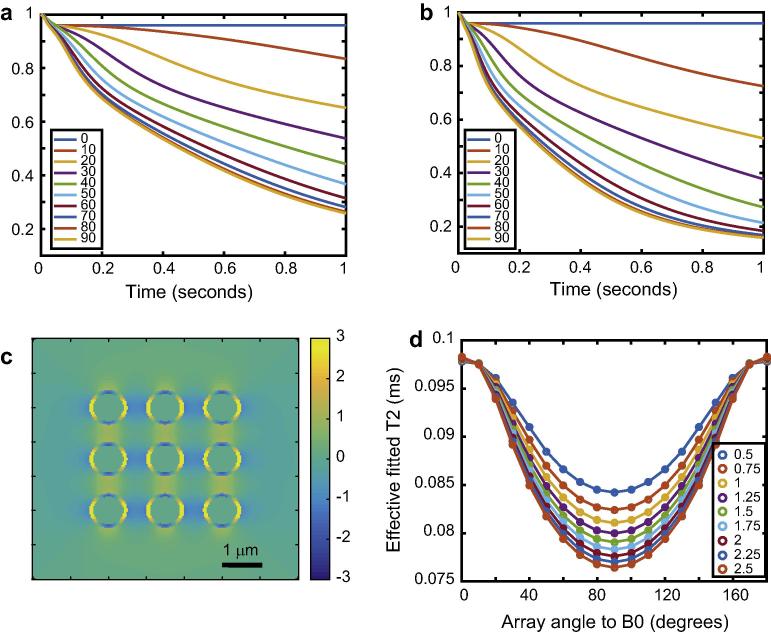
Effects of diffusion coefficient on nuclear spin phase decoherence in cylindrically porous media. Panels a and b show the signal lost due to decoherence by diffusion though magnetic field inhomogeneities created by the system’s geometry and susceptibility differences, with a corresponding to diffusion coefficient of 0.5 μm^2^/s and b to 2.5 μm^2^/s. Panel c shows the frequency difference map (with scale bar in Hz) with the system at 90° relative to *B*_0_. Panel d shows coherence lifetimes of effective *T*_2_ values again obtained by including a *T*_2_ of 0.1 s on the diffusion-related loss of coherence and fitting a mono-exponential function to the total decoherence function.

The effect of the diffusion coefficient was next investigated, in the range 0.5–2.5 μm^2^/s. The same system as in the previous simulations was used, expect that *B*_0_ was fixed at 3 T. The results are shown in Fig. 3. Although the same phenomenon may be seen as in the basic simulations of Fig. 1, we can also see that the total amount of anisotropy of coherence lifetime increases as the diffusion coefficient increases. This is rational, as spins experience a broader range of magnetic fields if they are able to move more rapidly. We also see that there is a non-zero phase decoherence even when the perfectly ordered array of cylinders is parallel to *B*_0_ if they have walls of finite thickness.

### 3.4 Effects of magnetic susceptibility difference

**Fig. 4:**
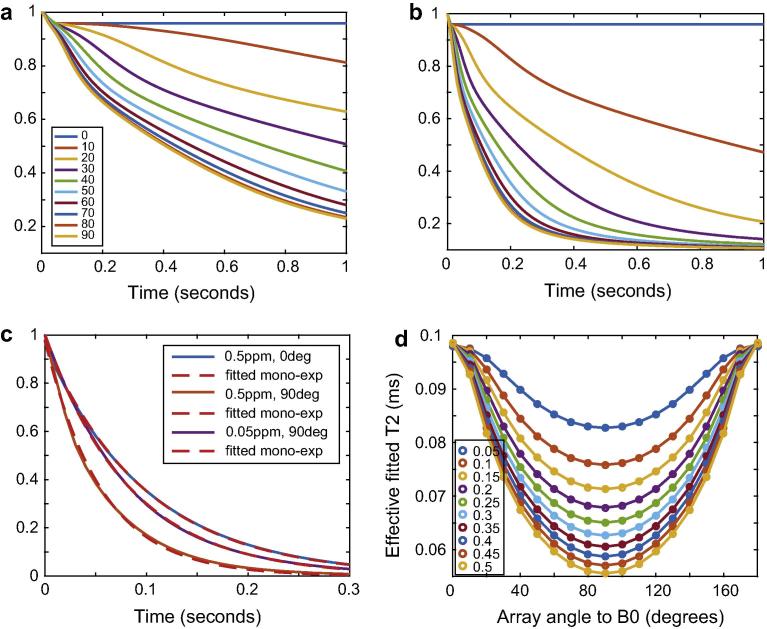
Effects of magnetic susceptibility difference on nuclear spin phase decoherence in cylindrically porous media. Panels a and b show the signal lost due to decoherence by diffusion though magnetic field inhomogeneities created by the system’s geometry and susceptibility differences, with susceptibility differences of 0.05 and 0.5 ppm respectively. Both panels share the legend in a. Panel c shows monoexponential fits to example signal decoherence functions defined as diffusion-mediated decoherence damped by exponential relaxation with a *T*_2_ relaxation time constant of 0.1 s. Panel d shows the coherence lifetimes generated by such means as a function of susceptibility difference and cylinder array angle relative to *B*_0_.

In our next simulations, we used the same system as in the previous section (whose geometry is shown in Fig.2c), but fixed the diffusion coefficient at 0.7 μm^2^/s and varied the magnetic susceptibility difference between the walls and lumen/surroundings. The results are shown in Fig. 4. Again, the form of the decoherence due to diffusion through field inhomogeneities is rather complicated but clearly “fastest” when the array of cylinders is inclined at 90° relative to *B*_0_ as seen in Fig.4a and b. We also include some examples of the quality of a mono-exponential fit to the exponentially damped decoherence functions inclusive of a *T*_2_ of 0.1 s in Fig.4c. The fits are of course not perfect but quite reasonable, vindicating the common use of an exponential coherence lifetime, including in our own work here. Panel 3d shows the coherence lifetime, with its familiar pattern of anisotropy, and with its anisotropy increasing as the susceptibility difference increases. With the particular geometry used, at 90° relative to *B*_0_, a susceptibility difference of 0.5 ppm has the effect that diffusion-mediated decoherence accounts for about half the observed coherence lifetime, which is correspondingly half the real *T*_2_.

### 3.5 Effects of order of cylindrical pores or objects

**Fig. 5:**
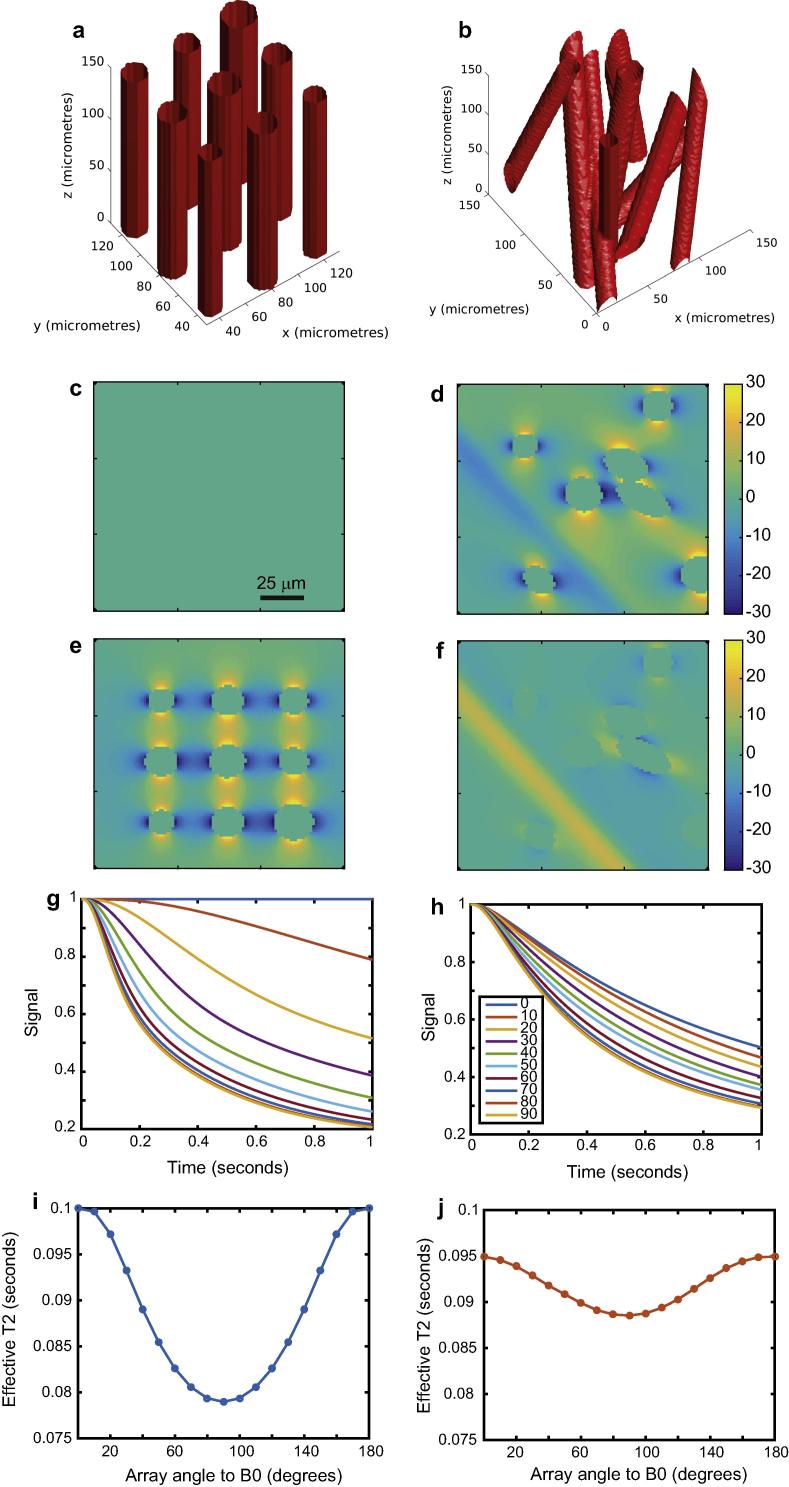
The effect of system order on nuclear spin phase decoherence in cylindrically porous media. Panels a and b show two different geometries of cylinders with infinitely thin, but impermeable, walls. Array a is perfectly aligned, but b has only a slight alignment parallel to its *z*-axis. Panels c and d show the frequency difference map at a representative slice through the geometries of a and b respectively when oriented at an angle of 0° relative to *B*_0_. Panels e and f show likewise but at 90° relative to *B*_0_. Panels g and h show the diffusion-mediated loss of signal for geometries a and b respectively. Scale bars are in units of Hz. Panels *i* and *j* show the coherence lifetime anisotropies for geometries a and b respectively inclusive of a *T*_2_ of 0.1 s. In all simulations the applied field was 3 T, the diffusion coefficient 0.7 μm^2^/s, the susceptibility difference 0.05 ppm.

Our next simulation examined the effects of how ordered the system of cylindrical field perturbers is upon the diffusion-mediated decoherence and coherence lifetime anisotropy. To do so, we set up two different arrays of hollow cylinders of infinitely thin but impermeable walls, and assumed a susceptibility difference between the lumen and surroundings. The first array comprised 9 cylinders of slightly different radii, all parallel and evenly spaced on a grid. The second array comprised the same 9 cylinders, but rotated and translated so as to retain a modest degree to alignment only, and not to lie on a regular grid. These geometries may be seen in Fig.5a and b. From panels c to f, we see that the disordered system always creates an inhomogeneous field; there is no angle at which the field vanishes. As such, there is always some dephasing in the disordered array. However, the dephasing also varies less as the system is rotated relative to *B*_0_, as seen in Fig.5g–j. The coherence lifetime anisotropy is greater overall in the ordered system, even though there is always some diffusion-mediated decoherence in the disordered system. This is since the fields produced by the disordered system may either interfere constructively or destructively. Often, cylinders at different angles conspire to cancel out the fields produced by one another to some extent in the disordered system, making the field gradients experienced by moving nuclear spins smaller than in the ordered system. A strong coherence lifetime anisotropy is therefore an indicator of a high degree of order.

### 3.6 The effects of wall thickness

**Fig. 6:**
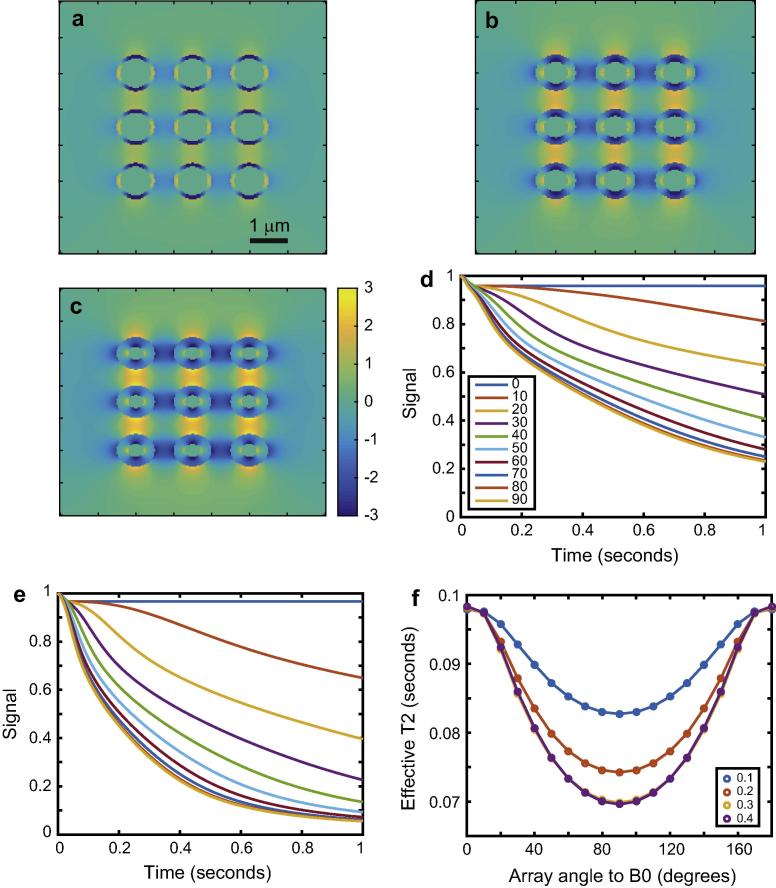
The effects of wall thickness on nuclear spin phase decoherence in cylindrically porous media. Panels a–c show the frequency difference maps (sharing the scale in in c, in Hz), at wall thicknesses of 0.1, 0.2 and 0.3 μm and a fixed outer radius of 0.5 μm. All maps are at an inclination of 90° to *B*_0_. Panels d and e show diffusion-mediated decoherence at wall thicknesses of 0.1 and 0.4 μm respectively. Panel f shows the coherence lifetimes inclusive of a *T*_2_ of 0.1 s by mono-exponential fitting. The *B*_0_ field was 3 T, the diffusion coefficient 0.7 μm^2^/s and the susceptibility difference 0.05 ppm.

We next simulated the effect of wall thickness in ordered arrays of cylinders in which the wall has different susceptibility from the lumen/surroundings. A similar system to that of Fig. 2, Fig. 3, Fig. 4 was used, save that the wall thickness was the variable and *B*_0_ fixed at 3 T. The results are shown in Fig. 6. The main result was the observation of increasing coherence lifetime anisotropy with wall thickness up to 0.3 μm. This is similar to the thickness of the myelin sheath *in vivo* [18]. As in all previous simulations, even with a relatively small susceptibility difference of 0.05 ppm and diffusion coefficient of 0.7 μm^2^/s, diffusion-mediated decoherence was a significant contributor to reducing coherence lifetime below *T*_2_ when the system of parallel cylinders was not parallel to *B*_0_. The fact that diffusion-mediated decoherence did not increase when the wall thickness increase beyond 0.3 μm (with a total radius of 0.5 μm) is probably because further increases do not add significantly to the total amount of wall material, adding as they do to the inner radius.

### 3.7 The effect of packing density

**Fig. 7:**
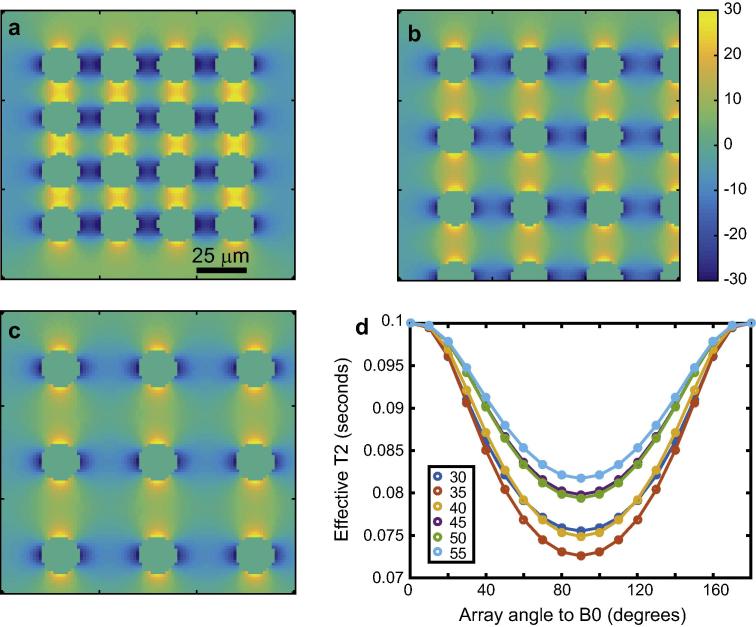
Effects of cylinder packing density on diffusion-mediated decoherence of nuclear spin phase. Panels a–c show frequency difference maps, all with the cylinder array inclined at 90° relative to *B*_0_ and sharing the scale bar of b, in Hz, for packing densities of 35, 40 and 50 μm respectively. The coherence lifetimes are plotted in panel d, showing a complex dependence on packing density.

Finally, we simulated the effects of cylinder packing density on diffusion-mediated decoherence by using arrays of cylinders of infinitely thin walls. Everything except the distance between cylinders (packing density) was kept constant. We performed simulations with packing densities from 30 to 55 μm with a fixed cylinder radius of 10 μm. The results are shown in Fig. 7. The coherence lifetime anisotropy shows the familiar pattern for each packing density. However, the dependence on packing density is not obvious. The packing density with the largest anisotropy of coherence lifetime was 35 μm, and whilst that with the smallest extent of coherence lifetime anisotropy was 55 μm, there is no obvious relationship. It is unlikely that coherence lifetime anisotropy could therefore be usefully deployed to infer information on packing density in practical situations.

## 4 Discussion

We have provided a formalism for describing diffusion-mediated decoherence of nuclear spin phase for systems with arbitrary magnetic field inhomogeneities and applied the formalism to the case of media containing cylindrical perturbers whose walls or lumen have different magnetic susceptibility from their surroundings. Such systems arise in cylindrically porous materials, but also in biological tissues abundant for instance in the brain. This can be considered of particular interest when interpreting MRI of the brain. In the white matter of the brain, bundles of closely aligned myelinated axons exist with a high degree of order, in which the myelin sheath around the axon has a distinct magnetic susceptibility from its surroundings. It is now accepted that the $T_{2}^{*}$, describing the coherence lifetime in a gradient-echo experiment without refocussing, is an anisotropic quantity in white matter predominantly because of the susceptibility difference between the myelin sheath and its surroundings [9], [11], [19], [20], [21], [22]. In many of our simulations, parameter choices have been driven by such a system. Although we stop some way short of claiming to have fully modelled this tissue type of the brain in our simple simulations, we may offer some insight. The capillary bed in white matter, where capillary lumens contain (varying amounts of) deoxyhaemoglobin, also has a high degree of alignment relative to the ordered axons [23], [24], [25] and provides another pertinent example of cylindrical field perturbers in biology. In structural biology, longitudinally stretched polyacrylamide gels are used to impose alignment on a system and thus measure its alignment tensor. Such a gel is elliptically porous, but the formalism here could be applied to aid in the description of relaxation in such systems.

The key results of our simulations are the following: first, diffusion-mediated decoherence of nuclear spin phase makes a substantial contribution to reducing the coherence lifetime below the *T*_2_ in ordered systems which are not parallel to *B*_0_, and disordered systems to a lesser extent but irrespective of orientation. Second, diffusion-mediated decoherence is anisotropic in ordered systems, having its most substantial effects when a system’s axis of order is perpendicular to *B*_0_. Third, the time-course of decoherence takes a complicated form, but to first order can be incorporated into relaxation as an exponential process. Finally, the extent of diffusion-mediated coherence increases with increasing diffusion coefficient and increasing absolute susceptibility difference.

We have seen in all our simulations that ordered systems display diffusion-mediated decoherence in an anisotropic fashion, with decoherence fastest when the system is perpendicular to the *B*_0_. We have also seen that the diffusion coefficient and susceptibility difference cause systematic alterations to the extent of diffusion-mediated decoherence. Therefore, coherence lifetime measurements, and in particular coherence lifetime anisotropy measurements, contain information on diffusion and susceptibility. This is of course apparent from the mathematics, but is represented more clearly by simulations in a simple but realistic system. A corollary is that measurements of susceptibility, diffusion and coherence lifetime may in future be combined to produce a consistent model for the system under examination. A conclusion based on one measurement type alone but which fails to predict another measurement type may not be acceptable.

Another corollary is the corruption of measurements of any particular parameter due to the failure to consider diffusion-mediated decoherence. As an example, consider an attempt to measure the translational diffusion tensor (or coefficient if isotropic) by a pulsed field-gradient NMR or MRI experiment. Such measurements always contain some small contamination from relaxation, but this is normally isotropic. However, if the decoherence due to cylindrical pores is especially severe, as our simulations show it to be at high magnetic fields, and for large susceptibility differences, one may introduce an additional decoherence due to the anisotropic local fields. This may in turn make the diffusion tensor a function of magnetic field strength and system orientation relative to magnetic field – even if the diffusion tensor were in reality isotropic.

### 4.1 Obtaining and using information from coherence lifetime measurements

The work we have presented deals only with predicting the effects of cylindrical field perturbers of known geometry and properties on nuclear spin phase decoherence, and we have thus far neglected the issue of garnering information about a system of unknown geometry and other parameters. We may envisage two main applications: the determination of geometric details of systems which are likely to possess cylindrical pores but which cannot be examined microscopically (such as biological tissues *in vivo*), and the removal of the effects of diffusion-mediated dephasing due to a known geometry to leave “pure” relaxation or pulsed field-gradient-mediated dephasing for further interpretation.

Although it is unlikely that the full geometric specification of a cylindrical array could be obtained by relaxation data alone, limiting-case models may be fitted to decoherence data, with spin-echoes sampled and a sufficient number of time points, and preferably with the availability of multiple orientations and magnetic fields, both of which are under experimental control, to at least some extent. “Limiting-case” models may be restricted to a regular array, for example, to extract an “effective pore radius”. Fitting may proceed by means of any number of techniques, though in many systems there is likely to be at least some prior information available so a Bayesian approach may be most effective. If the diffusion effects were to be removed, based on known geometry, one may again implement a model of reduced complexity. It is possible that data will be needed at multiple orientations and field strengths to garner quantitative information reliably and possibly with additional physical restraints, for example knowledge of the diffusion tensor or coefficient.

### 4.2 Relaxation due to translational diffusion through field inhomogeneities

We have made the distinction between decoherence and relaxation from the outset, and dealt only with decoherence. However, translational diffusion is a known relaxation mechanism, even if the magnetic field is homogeneous. Diffusion-mediated relaxation occurs since intermolecular dipolar (and quadrupolar for *I* > 1/2) couplings are stochastically modulated by translational diffusion. These effects have been studied, and are generally small compared to intramolecular dipolar and quadrupolar couplings (provided any exist). If the field is inhomogeneous, CSA becomes a relaxation mechanism since stochastic translations (even without rotations) alter the local field experienced by a nucleus. However, we consider the translational diffusion through inhomogeneous fields of the type presented in our simulations an unlikely candidate for a *relaxation* mechanism of any significance, and present our argument here. We might consider a simple model for relaxation due to translational diffusion through magnetic field inhomogeneities as follows: taking CSA as an example, for which the relevant basis functions are well known, the zero-order correlation function is

(21) $$C_{00}(t)=\frac{5}{9}\Delta^{2}\int (\omega_{0}+\Delta \omega (x))(\omega_{0}+\Delta \omega (x_{0}))\times (3\cos^{2}\theta -1)(3\cos^{2}\theta_{0}-1)P(x_{0})P(x\left|x_{0}\right))dxdx_{0}$$

Here, we have made the modification that the usual Larmor frequency is substituted for the Larmor frequency inclusive of the frequency difference function. The abbreviation Δ is loosely the anisotropy of the CSA tensor. The conditional probability $P(x\left|x_{0}\right))$, provided the media is isotropic, may be obtained using a random flight model, or a coarse approximation may be obtained simply by the translational diffusion equation. The latter case with a well-known solution leads to:

(22) $$C_{00}(t)=\frac{5}{9}\Delta^{2}\int (\omega_{0}+\Delta \omega (x))(\omega_{0}+\Delta \omega (x_{0}))\times (3\cos^{2}\theta -1)(3\cos^{2}\theta_{0}-1)P(x_{0}){\left(4\pi \mathrm{Dt}\right)}^{-\frac{3}{2}}\exp\left(-\frac{{\left(r-r_{0}\right)}^{2}}{4\mathrm{Dt}}\right)dxdx_{0}$$

Once equipped with a correlation function, calculation of relaxation time constants is straightforward. However, our simulations normally place the frequency difference in the range of tens of Hz, whereas the Larmor frequency is in the MHz range (128 MHz for 3 T). Given that translational diffusion is not a significant relaxation mechanism in most systems (though there are exceptions), a perturbation 6 orders of magnitude smaller than the Larmor frequency is unlikely to serve as a noticeable *relaxation* mechanism. The effects of small frequency differences due to magnetic field inhomogeneities are therefore likely to be manifest as nonlinear phase evolution and decoherence only. We can apply a similar argument to all other orders of the correlation function for CSA relaxation, and to dipolar relaxation. Where there exists an orienting potential, such as liquid crystals, rather more elaborate correlation functions are called for [26], [27]. In such systems, strong orienting potentials make translational diffusion a mechanism of *relaxation* anisotropy.

## 5 Conclusions

We have shown, by means of detailed simulations, that in systems with cylindrical pores whose walls or lumen are permeated by some media with a different magnetic susceptibility from the surroundings, that loss of nuclear spin phase coherence due to translational diffusion though the resulting local magnetic fields may be substantially more rapid than the effects of relaxation alone. The reduction of coherence lifetime below *T*_2_ is also anisotropic. An awareness of such phenomena is important when interpreting relaxation data, and may have utility in extracting greater information content from relaxation data.

## Appendix A Evaluation of spatial derivatives

We provide here some additional information on the evaluation of the gradients required to derive the equations describing diffusion though arbitrary magnetic fields. In the derivation, we come up against the equation:

(23) $$\nabla \cdot D\nabla [A\exp(i\rho \Delta \omega t)]=[\nabla \cdot D\nabla A+i\rho t\nabla A\cdot D\nabla \Delta \omega +A(i\rho t\nabla \cdot D\nabla -\rho^{2}t^{2}\nabla \Delta \omega \cdot D\nabla )\Delta \omega ]\exp(i\rho \Delta \omega t)$$

Here, we show some additional working. Firstly, note:

(24) $$\nabla \cdot D\nabla =\underset{j}{\sum }\underset{k}{\sum }\frac{\partial }{\partial x_{j}}D_{\mathrm{jk}}\frac{\partial }{\partial x_{k}}$$

Therefore:

(25) $$\nabla \cdot D\nabla [A\exp(i\rho \Delta \omega t)]=\underset{j}{\sum }\underset{k}{\sum }D_{\mathrm{jk}}\frac{\partial }{\partial x_{j}}\frac{\partial }{\partial x_{k}}A\exp(i\rho \Delta \omega t)$$

Now, apply the chain rule to obtain the following derivative:

(26) $$\frac{\partial }{\partial x_{k}}\exp(i\rho \Delta \omega t)=i\rho t\frac{\partial \Delta \omega }{\partial x_{k}}\exp(i\rho \Delta \omega t)=i\rho t\frac{\partial \Delta \omega }{\partial x_{k}}f$$

Next, apply the product rule to obtain:

(27) $$\begin{array}{c} \underset{j}{\sum }\underset{k}{\sum }D_{\mathrm{jk}}\frac{\partial }{\partial x_{j}}\frac{\partial }{\partial x_{k}}\mathrm{Af}=\frac{\partial^{2}A}{\partial x_{j}\partial x_{k}}f+\frac{\partial A}{\partial x_{k}}\frac{\partial f}{\partial x_{j}}+\frac{\partial A}{\partial x_{j}}\frac{\partial f}{\partial x_{k}}+A\frac{\partial^{2}f}{\partial x_{j}\partial x_{k}}\\ \;\;\;\;\;=\frac{\partial^{2}A}{\partial x_{j}\partial x_{k}}f+\frac{\partial A}{\partial x_{k}}i\rho t\frac{\partial \Delta \omega }{\partial x_{j}}f+\frac{\partial A}{\partial x_{j}}i\rho t\frac{\partial \Delta \omega }{\partial x_{k}}f+A\frac{\partial^{2}f}{\partial x_{j}\partial x_{k}} \end{array}$$

The final term of the RHS on the second line may be simplified according to:

(28) $$\begin{array}{c} \frac{\partial^{2}f}{\partial x_{j}\partial x_{k}}=\frac{\partial }{\partial x_{j}}\left[\mathrm{ipt}\frac{\partial \Delta \omega }{\partial x_{k}}f\right]\\ \;\;=\mathrm{ipt}\left[\frac{\partial^{2}\Delta \omega }{\partial x_{j}\partial x_{k}}f+\mathrm{ipt}\frac{\partial \Delta \omega }{\partial x_{j}}\frac{\partial \Delta \omega }{\partial x_{k}}f\right]\\ \;\;=\mathrm{ipt}\left[\frac{\partial^{2}\Delta \omega }{\partial x_{j}\partial x_{k}}+\mathrm{ipt}\frac{\partial \Delta \omega }{\partial x_{j}}\frac{\partial \Delta \omega }{\partial x_{k}}\right]f \end{array}$$

Such that:

(29) $$\underset{j}{\sum }\underset{k}{\sum }D_{\mathrm{jk}}\frac{\partial }{\partial x_{j}}\frac{\partial }{\partial x_{k}}\mathrm{Af}=\underset{j\text{,}k}{\sum }D_{\mathrm{jk}}\left(\frac{\partial^{2}A}{\partial x_{j}\partial x_{k}}+i\rho t\left[\frac{\partial A}{\partial x_{k}}\frac{\partial \Delta \omega }{\partial x_{j}}+\frac{\partial A}{\partial x_{j}}\frac{\partial \Delta \omega }{\partial x_{k}}\right]+A\left[i\rho t\frac{\partial^{2}\Delta \omega }{\partial x_{j}\partial x_{k}}-\rho^{2}t^{2}\frac{\partial \Delta \omega }{\partial x_{j}}\frac{\partial \Delta \omega }{\partial x_{k}}\right]\right)f=[\nabla \cdot D\nabla A+i\rho t[\nabla \Delta \omega \cdot D\nabla A+\nabla A\cdot D\nabla \Delta \omega ]+A(i\rho t\nabla \cdot D\nabla -\rho^{2}t^{2}\nabla \Delta \omega \cdot D\nabla )\Delta \omega ]\exp(i\rho \Delta \omega t)$$

Equipped with this, the full expression for the time derivative of ψ is:

(30) $$\frac{\partial }{\partial t}\psi =i\rho \Delta \omega A\exp(i\rho \Delta \omega t)+[\nabla \cdot D\nabla A+i\rho t[\nabla \Delta \omega \cdot D\nabla A+\nabla A\cdot D\nabla \Delta \omega ]+A(i\rho t\nabla \cdot D\nabla -\rho^{2}t^{2}\nabla \Delta \omega \cdot D\nabla )\Delta \omega ]\exp(i\rho \Delta \omega t)$$

However, we also have available the expression:

(31) $$\begin{array}{c} \frac{\partial }{\partial t}\psi =\frac{\partial }{\partial t}[A\exp(i\rho \Delta \omega t)]\\ \;=\frac{\partial A}{\partial t}\exp(i\rho \Delta \omega t)+\mathrm{Ai}\rho \Delta \omega \exp(i\rho \Delta \omega t) \end{array}$$

Equating the latter two formulae, then knocking out common factors and terms leads to the result in the main paper:

(32) $$\begin{array}{c} \frac{\partial A}{\partial t}=\nabla \cdot D\nabla A+i\rho t[\nabla \Delta \omega \cdot D\nabla A+\nabla A\cdot D\nabla \Delta \omega ]+A(i\rho t\nabla \cdot D\nabla -\rho^{2}t^{2}\nabla \Delta \omega \cdot D\nabla )\Delta \omega \\ \;=\exp(-i\rho \Delta \omega t)\underset{j}{\sum }\underset{k}{\sum }D_{\mathrm{jk}}\frac{\partial }{\partial x_{j}}\frac{\partial }{\partial x_{k}}A\exp(i\rho \Delta \omega t)\\ \;=\exp(-i\rho \Delta \omega t)\nabla \cdot D\nabla [A\exp(i\rho \Delta \omega t)] \end{array}$$
