# Corrigendum to “Diffusion-mediated nuclear spin phase decoherence in cylindrically porous materials”[J.Magn. Reson. 269 (2016) 1–12]

**DOI:** 10.1016/j.jmr.2016.09.017

**Published:** 2016-12

**Authors:** Michael J. Knight, Risto A. Kauppinen

**Affiliations:** a School of Experimental Psychology, University of Bristol, 12a Priory Road, Bristol, BS8 1TU, United Kingdom; b Clinical Research and Imaging Centre, University of Bristol, 60 St Michael’s Hill, Bristol, BS2 8DX, United Kingdom

The authors regret that the following funder acknowledgement was not clearly made: MJK was funded by an Elizabeth Blackwell Institute early career fellowship and by the Wellcome Trust International Strategic Fund ISSF2: 105612/Z/14/Z.

The authors would like to apologise for any inconvenience caused.

