# Supplementary material for: Diffusion-mediated nuclear spin phase decoherence in cylindrically porous materials
Source: J Magn Reson. 2016 Aug;269:1–12. doi: 10.1016/j.jmr.2016.05.007 (PMC4965358; doi:10.1016/j.jmr.2016.05.007)
Supplement: Supplementary data 1 [file mmc1.docx]

**Diffusion-mediated nuclear spin phase decoherence in cylindrically porous materials**

Michael J. Knight, Risto A. Kauppinen

**Supplementary Material**

**Validity of heuristic exponential time constants for diffusion attenuation**

In the main text, we demonstrate that decoherence of nuclear spin phase by translational diffusion through non-linear magnetic field inhomogeneities does not occur with the exponent of time or its second power. This is in contrast to solution-state relaxation in isotropic media, which is exponential, and translational diffusion through linear field gradients, which causes decoherence with the square of time. Nonetheless, we extract heuristic time constants, labelled coherence lifetimes, by combining our simulated diffusion attenuation due to inhomogeneous fields with isotropic relaxation, then fitting a mono-exponential function to that total decay. This emulates a realistic experiment, in which a simple function might be fitted to data sampled at a finite number of time points to return a heuristic coherence lifetime. However, the approach is model-dependent, so we explore here the possibility of using the second central moment of the signal decay, including and excluding a realistic rate of relaxation. This method is straightforward. The first moment is defined as:

$$\mu_{1}=\int_{-\infty}^{\infty} tS\left(t\right)dt$$

The second (central) moment is defined as:

$$\mu_{2}=\int_{-\infty}^{\infty} \left(t-\mu_{1}\right)^{2}S\left(t\right)dt$$

The square root of this quantity (the root-2^nd^ central moment) is then a time constant describing the “width” of the time-domain function *S*(*t*) and therefore is a model-free coherence lifetime describing how rapidly the function decays. However, since the integrals extend to infinity and both simulations and real data are necessarily truncated, this becomes a function of time sampling parameters. In reality, these terms are computed by numerical integration of a digitally sampled *t* and *S*(*t*).

To compare the relative stability of exponential time constants versus second central moments, we simulated diffusion attenuation using a model of multiple parallel cylindrical pores with walls of finite thickness, in which the walls were composed of a material with different magnetic susceptibility from the surroundings and lumen (the latter two regions having the same magnetic susceptibility). The simulations treated all regions as containing ^1^H-containing solvent to be observed. We created an array of 9 cylinders, inclined at 90° relative to B_0_, each of outer radius of 0.5 µm and inner radius of 0.1 µm. The susceptibility difference between the wall and surroundings/lumen was 0.05 ppm, the diffusion coefficient 0.7 µm^2^/s, and the B_0_ field 23 T, chosen to ensure substantial diffusion-mediated decoherence. This system was also used in some of the simulations of the main text Figure 2 (where the system may be visualised). In these stability tests, the simulation end time was varied from 0.1 to 1 s in increments of 0.1 s, but in all cases a resolution of 100 time points per simulation was used. A consistent number of points irrespective of simulation end time was necessary to ensure similar precision from the numerical (trapezoidal) integration used in the calculation of the 1^st^ and 2^nd^ moments.

The basic simulation results are plotted in Figure S1. Stability tests of exponential time constants and root-2^nd^ central moments are shown in Figure S2. The main results of the stability tests are that the relaxation-weighted exponential method, designed to emulated reality, yields very consistent results for simulation times of 0.2 s or more. The use of root-2^nd^ central moments has the advantage of being model-free, but results stabilise only if sampling of the decay is very thorough; inclusive of relaxation weighting, results stabilised only at simulation times > 0.5 s. Examining Figure S1, this requires sampling the time decay to a level of completion which in a real experiment would not be expedient; much of the data would be at or close to baseline noise level. However, the numerically stable exponential time constants remain model-dependent for a system which is not exponential, whereas the use of root-2^nd^ central moments has the advantage of being model-free.


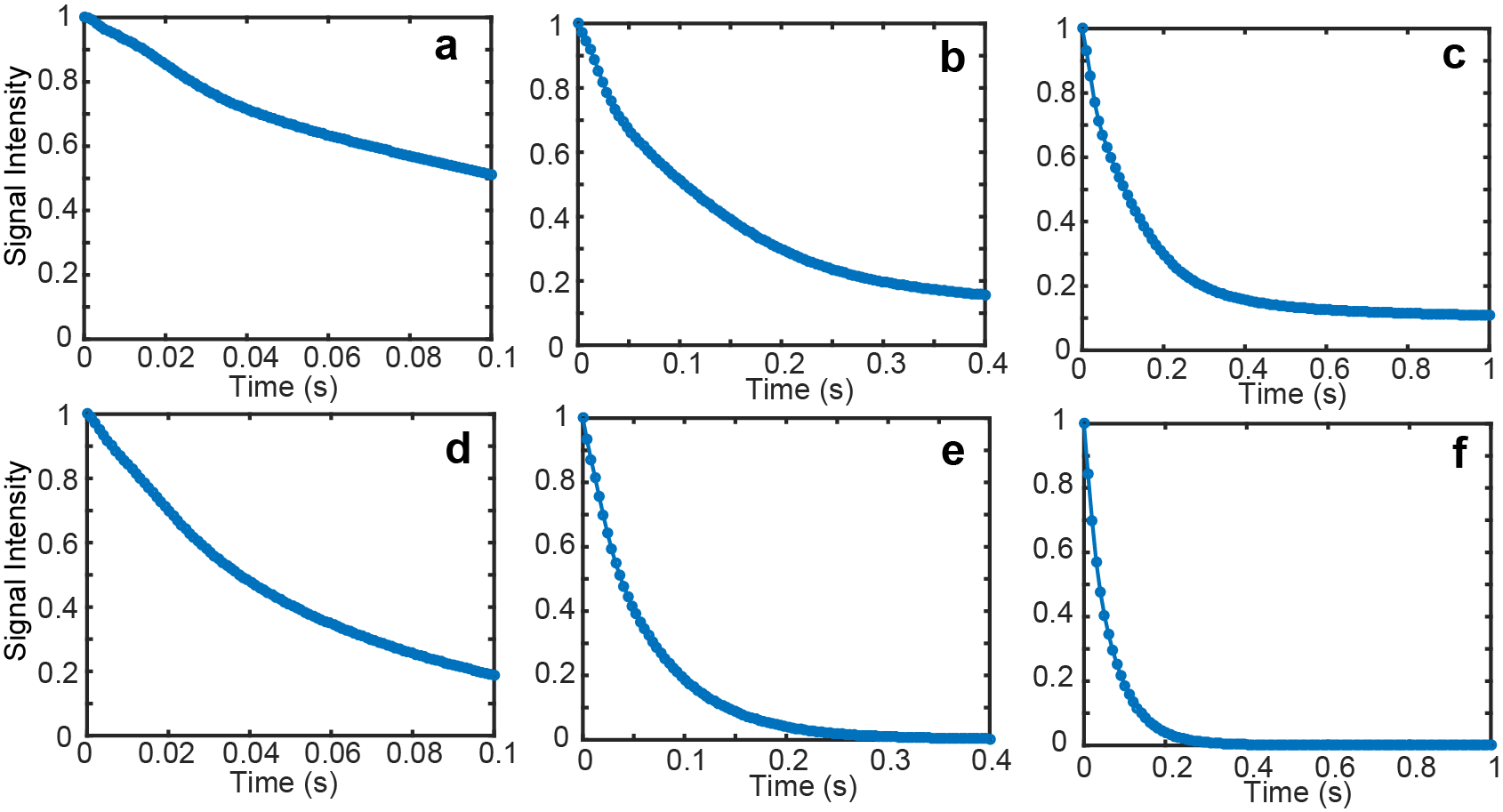


Figure S1: Diffusion-mediated decoherence with (a-c) and without (d-f) a 0.1 s isotropic exponential T_2_ weighting function, each with 100 time points but truncated at different final simulation times. Panels a-c show the “pure diffusion decoherence” effect whereas in d-f it is incorporated into the combined effects of relaxation and diffusion decoherence. The final simulation times are visible at the right of each x-axis.


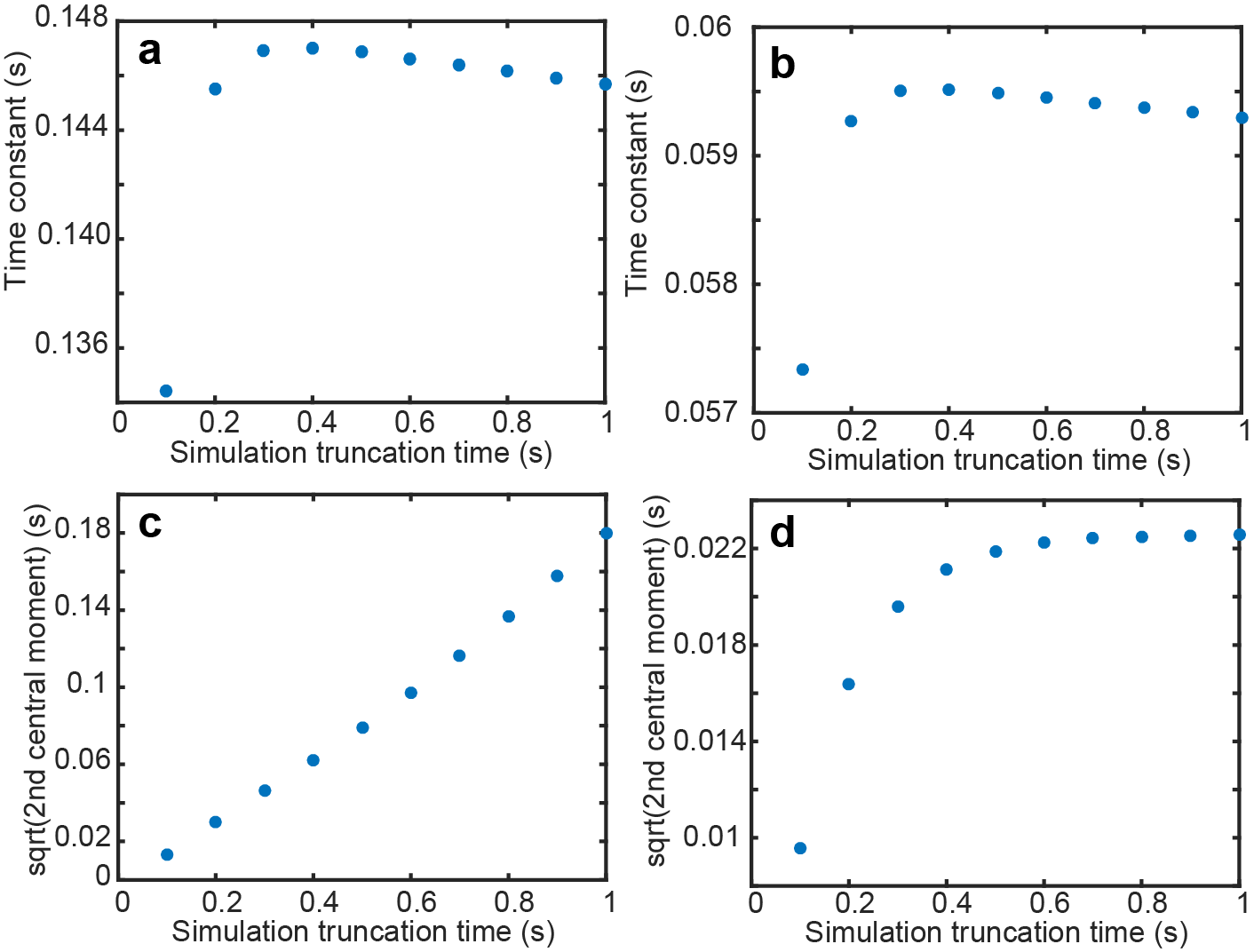


Figure S2: Stability of parameters describing the effects of diffusion-mediated decoherence. Different parameterisations of diffusion-mediated decoherence are plotted as a function of simulation truncation time, each using 100 time points in the simulation. Panel a shows mono-exponential time constants after removing the 0.1 s T_2_. Panel b shows the mono-exponential time constants inclusive of the 0.1 s T_2_. Panel c shows the root-2^nd^ central moment of the diffusion-mediated diffusion curves (without weighting by relaxation). Panel d shows the root-2^nd^ central moment of the diffusion-mediated diffusion curves inclusive of relaxation weighting. Note that the y-axis scales are different in each plot.
